# Refined Analysis of a Cross-Sectional Doping Survey Among Recreational Triathletes: Support for the Nutritional Supplement Gateway Hypothesis

**DOI:** 10.3389/fpsyg.2020.561013

**Published:** 2020-09-23

**Authors:** Sebastian Heller, Rolf Ulrich, Perikles Simon, Pavel Dietz

**Affiliations:** ^1^Institute of Occupational, Social and Environmental Medicine, University Medical Centre of the University of Mainz, Mainz, Germany; ^2^Department of Psychology, University of Tübingen, Tübingen, Germany; ^3^Department of Sports Medicine, Disease Prevention and Rehabilitation, Institute of Sports Science, University of Mainz, Mainz, Germany

**Keywords:** gateway, doping, nutritional supplements, triathletes, epidemiology

## Abstract

**Introduction:** The current literature provides no consensus that nutritional supplements (NS) may provide a gateway to doping. In particular, studies in recreational athletes are lacking. Within a previous cross-sectional empirical study, our group provided first evidence that the use of NS may provide a gateway for the use of doping substances in recreational triathletes. For the present paper, we refine the analysis of the triathletes’ survey in order to provide evidence for a NS gateway hypothesis in recreational athletes.

**Methods:** A self-report, paper-and-pencil questionnaire was distributed to a sample of 2,997 competitive ironman and half-ironman (*n* = 1,076; 36.1%) triathletes. The randomized response technique (RRT) was used to assess the 12-month prevalence estimate for the use of doping substances. The prevalence for the use of NS was assessed by using direct questioning. Two-tailed (*α* = 0.05) large-sample *z*-tests were performed to assess whether the estimated prevalence for the use of doping substances differs significantly between users and nonusers of NS.

**Results:** The 12-month prevalence estimate for the use of doping substances is significantly higher in athletes who report using NS (20.6%) compared to those who do not (11.4%; *z* = 2.595, *p* = 0.0097).

**Conclusion:** The present results are consistent with the hypothesis that the use of NS provides a gateway to the use of doping substances. Therefore, doping prevention concepts should not primarily focus on preventing the use of doping substances *per se*, but should start one step earlier, namely by the use of NS.

## Introduction

The World Anti-Doping Agency (WADA) defines doping “as the occurrence of one or more of the anti-doping rule violations” set forth in Articles 2.1–2.10 of the World Anti-Doping Code (World Anti-Doping Agency, 2019a). For example, these violations include the “presence of a prohibited substance or its metabolites or markers in an athlete’s sample,” “use or attempted use by an athlete of a prohibited substance or a prohibited method,” “possession of a prohibited substance or a prohibited method by an athlete or athlete support person” as well as “whereabouts rule violations” (World Anti-Doping Agency, 2019a). The authors of this paper focus on the first part of the doping definition referring to doping substance use. A comprehensive list of doping substances is given by WADA’s annually updated prohibited list (World Anti-Doping Agency, 2020).

A previous survey by the authors of the present paper indicates that almost 50% of the athletes who participated in two elite athletic competitions used doping substances during the previous 12 months (Ulrich et al., 2018). A more recent paper by Faiss et al. (2020) reveals a blood-doping prevalence between 15 and 18% among elite track and field athletes based on analyses of blood samples from two world athletics championships. These numbers support the results of a review on the prevalence of doping in adult elite athletes, summarizing studies based on different methods for pharmacological and biological parameters as well as questionnaires to assess doping, in which estimates for the prevalence of doping between 14 and 39% are reported (de Hon et al., 2015).

The use of doping substances is not only common among elite athletes but also reported for recreational athletes. For example, a survey among 800 amateur athletes and exercisers by Lazuras et al. (2017) reveals a lifetime prevalence for doping of 18.3%. Furthermore, doping past-year prevalence estimates of 6.5% (Molero et al., 2017) and 8.2% (Stubbe et al., 2014) as well as lifetime prevalence estimates of 12.5% (Simon et al., 2006) and 14.0% (Mooney et al., 2017) are reported in fitness center members. For recreational endurance athletes, prevalence estimates for doping of 8.1% (Locquet et al., 2017) and 8.4% (Campian et al., 2018) are reported.

From a public health point of view, the abovementioned prevalence estimates for the use of doping substances, especially in recreational athletes, are alarming. The use of doping substances appears to be associated with physiological (e.g., cardiovascular, metabolic, endocrine, hepatic, renal, and musculoskeletal) and psychological side effects and with an increased mortality in general (Pope et al., 2014; Momaya et al., 2015; Cantelmo et al., 2019; Atkinson and Kahn, 2020). However, understanding why athletes and especially recreational athletes tend to use doping substances contributes to evidence-based planning of anti-doping interventions because effective programs have to target factors causally related to the use of doping substances. Therefore, it is necessary to investigate potential correlates (factors that are associated with the use of doping substances) or determinants (factors with a causal relationship) of doping (Miettinen, 2010). In this context, a meta-analysis by Ntoumanis et al. (2014) shows that perceived social norms and positive attitudes toward doping might predict doping behavior. In addition, a recent study by Tavares et al. (2019) indicates that attitudes, beliefs, and especially subjective norms predict the intention to use doping substances among gym users.

Among the different factors that might be associated with the use of doping substances, the use of nutritional supplements (NS; Garthe and Maughan, 2018) is also discussed to predict the (self-reported) use of doping substances or, in other words, to provide a gateway (Kandel, 2002) to doping (Papadopoulos et al., 2006; Hildebrandt et al., 2012; Backhouse et al., 2013; Barkoukis et al., 2019). For example, within their review, Garthe and Maughan (2018) state that there is “some data to suggest that supplement users have more positive attitudes toward doping (…).” In this context, a meta-analysis by Ntoumanis et al. (2014) shows that the use of NS might predict doping intentions and doping behaviors among athletes. However, this part of the meta-analysis is based on only three studies.

In conclusion, there is no consensus in the current literature that NS might predict the use of doping substances among recreational athletes. Within a cross-sectional empirical study, our group provides first evidence that the use of NS might provide a gateway for the use of doping substances in recreational triathletes (Dietz et al., 2013b). This previous study employs the randomized response technique (RRT) to estimate the prevalence for the use of doping substances. One strength of this indirect survey technique is that it protects respondents with regard to socially sensitive issues (Lee and Renzetti, 1990; Dietz et al., 2018b), resulting in more valid prevalence estimates for the sensitive item compared to direct questioning (Lensvelt-Mulders et al., 2005). However, using this technique, the prevalence for the socially sensitive issue – in our case, the use of doping substances – can only be assessed for the whole collective and not for a single individual. Consequently, our previous assumption that NS may provide a potential gateway to the use of doping substances is based on descriptive analysis (group differences) and bootstrapping. For the present paper, we refine the analysis of the triathletes’ survey using large-sample *z*-tests in order to provide a stronger evidence base regarding a potential gateway from NS to the use of doping substances in recreational athletes.

## Materials and Methods

A short, self-report, paper-and-pencil questionnaire addressing the use of performance-enhancing substances was distributed to a sample of 2,997 competitive ironman and half-ironman triathletes during registration in the race offices of three ironman competitions in Germany (Frankfurt, Regensburg, and Wiesbaden 70.3). The unrelated question model (UQM), a version of the RRT (Dietz et al., 2013a,b, 2018a,b; Franke et al., 2013, 2017; Schröter et al., 2016), is used to assess the 12-month prevalence estimate for the use of doping substances. The prevalence for the use of NS is assessed by using direct questioning. Statistical power analysis, according to Ulrich et al. (2012), was performed *a priori* to determine sample size. The null hypothesis of this power analysis assumes that the true prevalence $\left({\hat{\pi }}_{s}\right)$ is equal to zero. To detect an overall prevalence of at least 6% with a statistical power of *p* = 0.85 and given that, according to previous competitions, only one quarter of the athletes were female, the sample size *n* should be at least equal to 2,600 so that the RRT yields meaningful results even for the subsample of females. The athletes gave written consent to participate in the survey within the questionnaire. Ethical approval to conduct this study was obtained by the Eberhard Karls University of Tübingen Ethics Committee. For further information on the methodology, please find a detailed description of the study in the original paper (Dietz et al., 2013b). A numerical example of how to estimate the prevalence for a sensitive item$\left({\hat{\pi }}_{s}\right)$ with the UQM is given in Franke et al. (2013). Prevalence estimates for the use of doping substances $\left({\hat{\pi }}_{s}\right)$ are presented as percentages with 95% confidence intervals (CI) and standard error and were computed using Matlab version R2015a.

For the present paper, two-tailed (*α* = 0.05) large-sample *z*-tests (Dietz et al., 2018a) were performed to assess whether the estimated prevalence for the use of doping substances differs significantly between users and nonusers of NS. Therefore, we used R software version 3.2.3.

## Results

A total of 2,987 recreational triathletes (response rate 99.7%) returned the anonymous questionnaire. The percentage of valid responses to the RRT part (use of doping substances) was 90.5% (*n* = 2,702). Most of the athletes were male (*n* = 2,576, 87.3%), and the mean age was 39.5 years (*SD*: ± 9.2; range 18–79).

The overall estimated 12-month prevalence for the use of doping substances is 13.0%. Two-tailed large sample *z*-tests reveal that the prevalence estimate for the use of doping substances is significantly higher among users of NS (20.6%) compared to nonusers (11.4%; *z* = 2.595, *p* = 0.0097; Table 1).

**Table 1:** Estimated 12-month prevalence for physical doping using the unrelated question model (UQM).

Variable	Yes	No	*a*	${\hat{\pi }}_{s}$ (%)	*SE(*${\hat{\pi }}_{s}$*)* (%)	95% CI	*Z*; *p*
All athletes (*n* = 2,702)^*^	676	2,026	0.250	13.0	1.2	10.5–15.4	
Nutritional supplement use							2.595; 0.0097
Yes	115	267	0.301	20.6	3.5	13.7–27.4	
No	542	1,723	0.239	11.4	1.3	8.7–14.0	

* Of the 2,987 athletes that filled in the questionnaire, 285 athletes provided no valid response on the RRT question resulting in a case number of 2,702 for the present table.

${\hat{\pi }}_{s}$, prevalence estimate for the use of doping substances; *a*, the proportion of total “yes” responses in the sample.

## Discussion

The present paper aims to provide stronger evidence regarding the NS gateway hypothesis in recreational athletes by presenting a refined analysis of a previously performed doping survey among recreational triathletes. Specifically, a two-tailed, large-sample *z*-test reveals that the 12-month prevalence estimate for the use of doping substances is significantly higher in athletes who report using NS compared to athletes who deny this question. Although cross-sectional designs cannot directly assess causality, the significant association between the use of NS and the use of doping substances is nevertheless consistent with the notion that NS provide a gateway to the use of doping substances among recreational triathletes. This gateway hypothesis is also supported by the qualitative study from Lentillon-Kaestner and Carstairs (2010) using anonymous semi-structured interviews performed among a sample of elite athletes. The participants of this former study state that the use of NS was the first step to the use of doping substances. In recreational athletes, the same process is plausible, but further studies, especially longitudinal as well as qualitative approaches focusing on the chronology of consumed substances, are lacking.

From a public health point of view, it is important to identify potential factors that predict the use of doping substances in order to develop evidence-based anti-doping interventions. In particular, effective programs have to target factors that cause the use of doping substances (Miettinen, 2010; Ntoumanis et al., 2014). For example, the review by Morente-Sánchez and Zabala (2013) shows that most athletes who report using doping substances do this although they are aware of the risks on sanctions and health. Thus, purely educating interventions on the use of doping substances may be less effective. Consequently, given that the present results support the hypothesis that the use of NS may provide a gateway to the use of doping substances, doping prevention concepts should not primarily focus on preventing the use of doping substances, but should start one step earlier, namely by the use of NS. In this context, athletes, and especially young athletes, should learn to use NS according to their individual physiological requirements and not according to the motto “the more, the better.” Furthermore, even the use of NS may entail adverse health consequences as some studies report that a noticeable percentage of supplements are polluted or contain prohibited substances without labeling (Kohler et al., 2010; Maughan et al., 2018). In addition, NS quality tests are executed infrequently by regulating authorities in many nations (Molinero and Márquez, 2009; Outram and Stewart, 2015; Garthe and Maughan, 2018; Maughan et al., 2018). Hence, a practical application resulting from the present study is to put more effort into coaches’ and athletes’ education regarding the use of NS, especially with focus on young athletes. Due to the potential risks of using NS (doping and health risk through pollution and acting as a gateway to doping), coaches and athletes should treat NS in a more careful way, for example, using NS only in consultation with a sports physician and not according to the motto “the more, the better.” A concrete example of education on the risks of NS use is already included in the “parents’ guide to support clean sport” by World Anti-Doping Agency (2019b).

To conclude, based on the results of the present study, in recreational athletes, NS may provide a potential gateway to the use of doping substances and should not be seen as a safe alternative. Strengths of the study are the large sample of triathletes and the use of the RRT to estimate the prevalence of doping as well as the high response rate. However, it has to be stressed that the results of the present study are limited to the specific population of ironman and half-ironman triathletes. In other populations, a potential gateway from NS to doping needs to be confirmed by future studies. Furthermore, research from the field of behavioral science shows that it might be important to identify the influencing factors behind the behavior of the use of NS and doping substances to implement appropriate prevention strategies at the right time in the athletic career (Petróczi and Aidman, 2008). In this context, motives and intentions behind the use of NS and also behind shifting toward doping are unknown and need to be explored before more tailored prevention interventions can be planned (Backhouse et al., 2013). Possible approaches to investigate the moderating role of achievement goals and motivation (Barkoukis et al., 2019) or self-regulatory efficacy (Boardley et al., 2017) could lead to new intervention strategies in future.

## Data Availability Statement

The raw data supporting the conclusions of this article will be made available by the authors, without undue reservation.

## Ethics Statement

The studies involving human participants were reviewed and approved by Eberhard Karls University of Tübingen Ethics Committee. The patients/participants provided their written informed consent to participate in this study.

## Author Contributions

All authors contributed to the conception, analysis, and interpretation of the manuscript. All authors read and approved the final document.

### Conflict of Interest

The authors declare that the research was conducted in the absence of any commercial or financial relationships that could be construed as a potential conflict of interest.
